# Improvement of putrescine production through the arginine decarboxylase pathway in *Escherichia coli* K-12

**DOI:** 10.1186/s13568-021-01330-5

**Published:** 2021-12-15

**Authors:** Kullathida Thongbhubate, Kanako Irie, Yumi Sakai, Akane Itoh, Hideyuki Suzuki

**Affiliations:** Division of Applied Biology, Kyoto Institute of Technology, Matsugasaki, Sakyo-Ku, Kyoto, 606-8585 Japan

**Keywords:** Glutamate-putrescine ligase, *N*-acetylglutamate synthase, Macrodomain Ori protein, Terrific broth

## Abstract

In the bio-based polymer industry, putrescine is in the spotlight for use as a material. We constructed strains of *Escherichia coli* to assess its putrescine production capabilities through the arginine decarboxylase pathway in batch fermentation. *N*-Acetylglutamate (ArgA) synthase is subjected to feedback inhibition by arginine. Therefore, the 19th amino acid residue, Tyr, of *argA* was substituted with Cys to desensitize the feedback inhibition of arginine, resulting in improved putrescine production. The inefficient initiation codon GTG of *argA* was substituted with the effective ATG codon, but its replacement did not affect putrescine production. The essential genes for the putrescine production pathway, *speA* and *speB*, were cloned into the same plasmid with *argA*^ATG Y19C^ to form an operon. These genes were introduced under different promoters; *lacI*p, *lacI*^q^p, *lacI*^q1^p, and T5p. Among these, the T5 promoter demonstrated the best putrescine production. In addition, disruption of the *puuA* gene encoding enzyme of the first step of putrescine degradation pathway increased the putrescine production. Of note, putrescine production was not affected by the disruption of *patA,* which encodes putrescine aminotransferase, the initial enzyme of another putrescine utilization pathway. We also report that the strain KT160, which has a genomic mutation of YifE^Q100TAG^, had the greatest putrescine production. At 48 h of batch fermentation, strain KT160 grown in terrific broth with 0.01 mM IPTG produced 19.8 mM of putrescine.

**Supplementary Information:**

The online version contains supplementary material available at 10.1186/s13568-021-01330-5.

## Introduction

Putrescine is a polyamine and consists of two amino groups and four methylene groups. This compound is widely distributed in living organisms (Pegg 1986; Michael 2016; Keller et al. 2019). Several studies have focused on putrescine as it regulates rapid cell proliferation and differentiation at the level of gene expression (Heby 1981; Michael 2018; Igarashi and Kashiwagi 2000, 2018, 2019). Yoshida et al. (2004) reported a correlation between putrescine and more than 600 genes related to the regulation of transcription. In addition, putrescine is used as a precursor of other polyamines, surfactants, and agrochemicals, and as a component of polymers such as nylon 4,6 (Schneider and Wendisch 2011; Wendisch et al. 2018; Hui et al. 2020). The polycondensation of putrescine and adipic acid is used to synthesize nylon 4,6. Due to its flexibility and high solvent resistance properties, nylon 4,6 was introduced into the commercial field (Demco et al. 2007; Yamanobe et al. 2007). Polyamides are used in the electric vehicle industry as materials to develop a lighter body, interior, motor, controller, and electronic board of the car. Along with the expansion of the global electric vehicle market, the demand for putrescine is also increasing (Scott et al. 2007; Schneider and Wendisch 2010). There are two processes to synthesize putrescine, chemical and biological. However, the chemical process producing an intermediate, succinonitrile, releases dangerous and harmful compounds. On the other hand, the biological process is safe, environmentally friendly, and uses renewable feedstock (Sanders et al. 2007; Nguyen and Lee 2019; Li et al. 2020a).

**Fig. 1:**
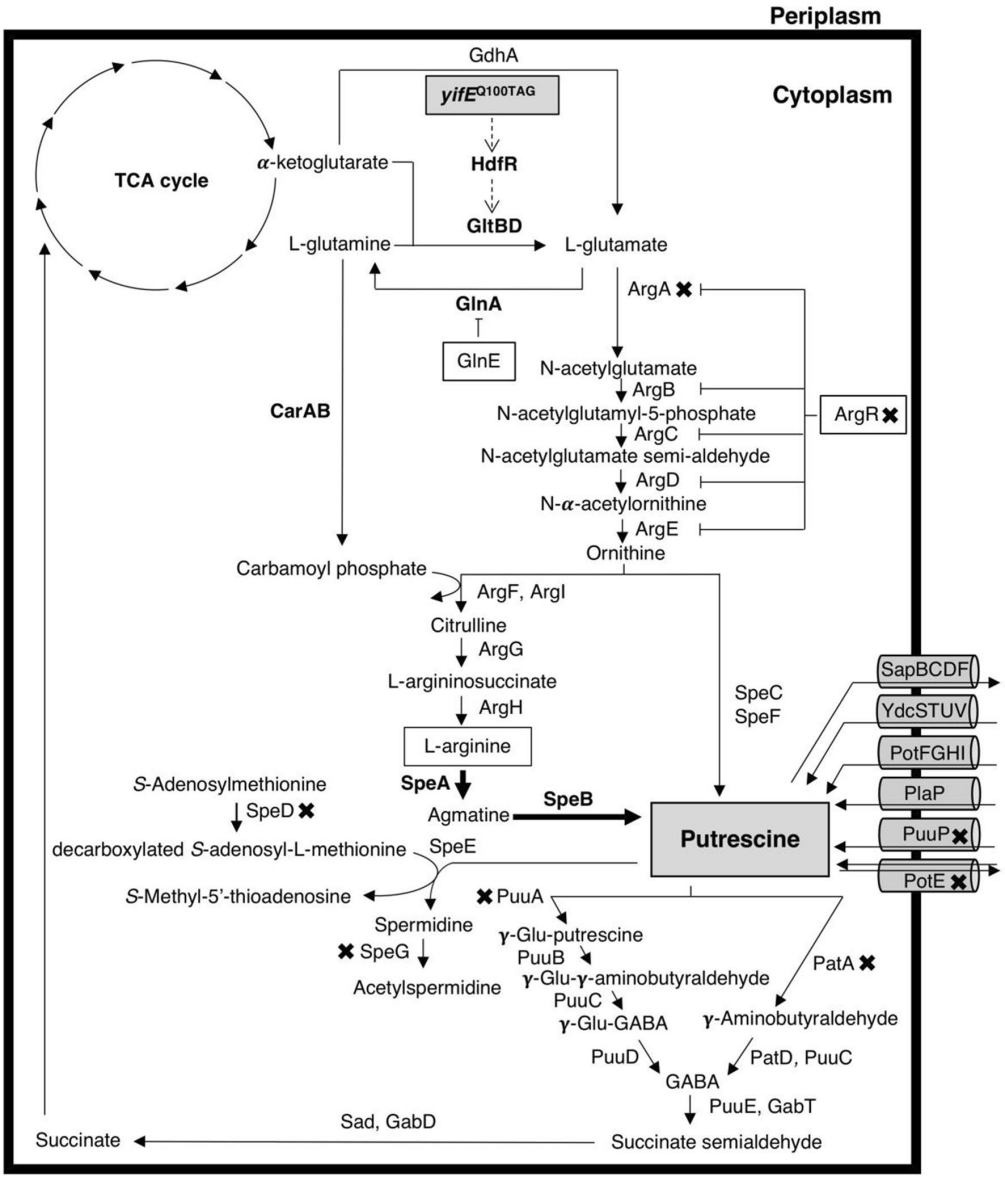
The metabolic map of putrescine in *E. coli* K-12. The Xs indicate the knocked out genes. Thick arrows indicate the overexpression of genes. Dash arrows indicate the increased expression of genes. Blunt end arrows indicate inhibition of the expression of genes. *GdhA* glutamate dehydrogenase, *HdfR* DNA-binding transcriptional dual regulator HdfR, *GltBD* glutamate synthase, *GlnA* glutamine synthetase, *GlnE* glutamine synthetase adenylyltransferase, *CarAB* carbamoyl phosphate synthetase, *ArgA* *N*-acetylglutamate synthase, *ArgB* acetylglutamate kinase, *ArgC* *N*-acetylglutamylphosphate reductase, *ArgD* *N*-acetylornithine aminotransferase, *ArgE* acetylornithine deacetylase, *ArgF* ornithine carbamoyltransferase, *ArgI* ornithine carbamoyltransferase, *ArgG* argininosuccinate synthetase, *ArgH* argininosuccinate lyase, *ArgR* DNA-binding transcriptional dual regulator ArgR, *SpeA* biosynthetic arginine decarboxylase, *SpeB* agmatinase, *SpeC* constitutive ornithine decarboxylase, *SpeD* *S*-adenosylmethionine decarboxylase, *SpeE* spermidine synthase, *SpeF* inducible ornithine decarboxylase, *SpeG* spermidine *N*-acetyltransferase, *PatA* putrescine aminotransferase, *PatD* γ-aminobutyraldehyde dehydrogenase, *GabT* 4-aminobutyrate aminotransferase, *GabD* succinate-semialdehyde dehydrogenase GabD, *Sad* succinate-semialdehyde dehydrogenase Sad, *PuuA* glutamate-putrescine ligase, *PuuB* γ-glutamylputrescine oxidase, *PuuC* γ-glutamyl-γ-aminobutyraldehyde dehydrogenase, *PuuD* γ-glutamyl-γ-aminobutyrate hydrolase, *PuuP* putrescine importer, *YdcSTUV* putrescine importer, *PotFGHI* putrescine importer, *PlaP* putrescine importer, *PotE* putrescine-ornithine antiporter/putrescine importer, *SapBCDF* putrescine exporter, *TCA cycle* tricarboxylic acid cycle

Among the living organisms that can produce putrescine, bacteria are commonly used in research because they grow rapidly under simple culture conditions such as *Escherichia coli*. The knowledge of polyamines in *E. coli* was reviewed admirably by Tabor and Tabor (1985) a long time ago, but it is still valid today. *E. coli* has two putrescine synthetic pathways (Fig. 1): the ornithine decarboxylase (ODC) pathway and the arginine decarboxylase (ADC) pathway, which use ornithine and arginine as starting compounds, respectively (Fig. 1). Arginine is synthesized from glutamate via ornithine through a sequential reaction. The ODC pathway decarboxylates ornithine to putrescine by constitutive ornithine decarboxylase (SpeC) and/or inducible ornithine decarboxylase (SpeF) (Tabor and Tabor 1985). The other pathway, the ADC pathway, converts arginine to putrescine via agmatine. The conversion of arginine to agmatine is catalyzed by biosynthetic arginine decarboxylase (SpeA) and biodegradative arginine decarboxylase (AdiA). Subsequently, agmatine is hydrolyzed to putrescine by agmatinase (SpeB). Putrescine is catabolized by two pathways, the putrescine utilization pathway (Puu pathway) (Kurihara et al. 2005) and the putrescine aminotransferase (PatA)-γ-aminobutyraldehyde dehydrogenase (PatD) pathway. These catabolic pathways rely on glutamate-putrescine ligase (PuuA) and putrescine aminotransferase (PatA), respectively (Samsonova et al. 2003, 2005; Kurihara et al. 2008). *S*-Adenosylmethionine decarboxylase (SpeD) generates decarboxylated *S*-adenosyl-L-methionine. Its *n*-propylamine is transferred to putrescine via spermidine synthase (SpeE) to synthesize spermidine (Tabor et al. 1986). Spermidine *N*-acetyltransferase (SpeG) converts spermidine to its inactive form, *N*-acetylspermidine (Limsuwun and Jones, 2000). In addition, Haywood and Large (1985) reported that *speG* encodes a putative diamine acetyltransferase that converts putrescine to acetylputrescine in *Candida boidinii*. Although the use of putrescine by *E. coli* SpeG as a substrate has not been reported, the *speG* gene was knocked out in this study to prevent the possible loss of putrescine (Fig. 1). Because of the cationic function of polyamine, it cannot penetrate through the cell membrane. Two spermidine transporters, PotABCD and MdtJI, and six putrescine transporters, PuuP, YdcSTUV, PlaP, PotE, PotFGHI, and SapBCDF, have been identified (Kashiwagi et al. 1992; Pistocchi et al. 1993; Kurihara et al. 2009a, b; Kurihara et al. 2011; Saier et al. 2016; Sugiyama et al. 2016). We recently reported that PotFGHI can import spermidine under biofilm-forming conditions (Thongbhubate et al. 2021).

Qian et al. (2009) overproduced putrescine through the ODC pathway in *E. coli* K-12, and the batch culture experiment resulted in 1.68 g/L (19.0 mM) in the 10 g/L of the glucose-enriched medium. Recently, Li et al. (2020b) overexpressed the ADC pathway and improved putrescine production using *E. coli* BL21(DE3). They produced putrescine using an SOB medium supplemented with 8 g/L of glucose and 12 g/L of arginine, resulting in 4.77 g/L of putrescine in batch culture. However, the latter method is a type of sequential enzymatic conversion of arginine to putrescine.

We found that the introduction of the plasmid that contains *speAB* produced more extracellular putrescine than that of the plasmids that contain *speC* or *speABC*. Therefore, we focused on fermentation of putrescine via the ADC pathway of *E. coli* K-12 without the addition of arginine. Here, genetic engineering was used as a tool to increase the production of putrescine. ArgA catalyzes the initial step of arginine biosynthesis (Rajagopal et al. 1998; Shin and Lee 2014; Ginesy et al. 2015). When arginine is overproduced, this compound affects *N*-acetylglutamate synthase (ArgA) activity through negative feedback inhibition. Furthermore, the DNA-binding transcriptional dual regulator (ArgR) represses the transcription of the genes of *arg* regulons, including *argA*. We not only attempted to enhance arginine synthesis, but also deleted genes related to the degradation or utilization of putrescine, in addition to transporters that uptake putrescine from the medium, such as *potE*, *speG*, *patA*, *speD*, *argR* and *puuPA*, and altered the expression of genes related to the synthesis of putrescine, *speA*, *speB*, and other genes from plasmids (Fig. 1).

## Materials and methods

### Construction of *E. coli* strains

The *E. coli* strains used in this study are listed in Table 1. The chromosomal genes *speD,* and *argR* were replaced with FRT (FLP recombination target)-*kan*^R^-FRT from the Keio gene knockout collection (Baba et al. 2006) by P1*vir* transduction (Miller 1972). The disruption of *speG*, *potE,* and the ATP binding site of the *puuA* gene were described previously (Kurihara et al. 2008, 2009b; Thongbhubate et al. 2021). The *patA* and *potFGHI* genes were disrupted by a method described previously (Datsenko and Wanner 2000) using pKD13 as a template for the PCR amplification with delta-ygjG F and delta-ygjG R as primers for *patA*, and potF-up and potI-down as primers for *potFGHI* (Additional file 1: Table S1). The *kan*^R^ genes were eliminated from FRT-*kan*^R^-FRT by Flp flippase carried by pCP20 (Datsenko and Wanner 2000). To confirm the deletion of genes, colony PCR was performed with primers (Additional file 1: Table S1) that annealed upstream and downstream of the genomic regions of the target genes, and the sizes of the amplicons were measured. To construct SH2204, the *yifE*::FRT-*kan*^R^-FRT of SH2201 was replaced with the *yifE*^Q100TAG^ using the *zie-296*::Tn*10* as a co-transduction marker by P1*vir* phage grown on SH2203 to obtain SH2204, which was confirmed to be Kan^S^. Then, the *yifE* region of SH2204 was amplified by PCR and the DNA sequence was confirmed. For DNA modification and strain construction, *E. coli* cells were grown in LB medium (Miller 1972). Where appropriate, culture media were supplemented with 30 μg/mL of kanamycin, 100 μg/mL of ampicillin, or 15 μg/mL of tetracycline. For the pre-cultures, 10 mL of LB medium was inoculated with a single colony in 100-mL Erlenmeyer flasks. Cultures were incubated at 37 °C with reciprocal shaking at 120 rpm.

**Table 1:** Strains used in this study

Strains	Genotype	Source or reference
FS113	MG1655 except Δ*argR*::FRT-*kan*^R^-FRT	This study
	Δ*patA*::FRT Δ*potE*::FRT Δ*speD*::FRT	
	Δ*speG*::FRT Δ*puuA*::FRT	
FS115	MG1655 except Δ*argR*::FRT	This study
	Δ*patA*::FRT Δ*potE*::FRT Δ*speD*::FRT	
	Δ*speG*::FRT Δ*puuA*::FRT	
FS123	pSH1734/FS113	This study
MG1655	F^−^ *rph*-*1*	C. A. Gross
KN20	pKN18/FS113	This study
KN24	pKN11/FS113	This study
KT32	FS115 except Δ*argA*::FRT-*kan*^R^-FRT	This study
KT37	pSH1823/KT32	This study
KT38	pSH1807/KT32	This study
KT39	pSH1820/KT32	This study
KT43	MG1655 except Δ*argR*::FRT	This study
	Δ*patA*::FRT Δ*potE*::FRT Δ*speD*::FRT	
	Δ*speG*::FRT Δ*argA*::FRT	
	Δ*puuPA*::FRT-*kan*^R^-FRT	
KT105	MG1655 except Δ*argR*::FRT	This study
	Δ*patA*::FRT Δ*potE*::FRT Δ*speD*::FRT	
	Δ*speG*::FRT Δ*argA*::FRT-*kan*^R^-FRT	
Strains	Genotype	Source or reference
KT112	MG1655 except Δ*argR*::FRT	This study
	Δ*potE*::FRT Δ*speD*::FRT Δ*speG*::FRT	
	Δ*puuPA*::FRT Δ*argA*::FRT-*kan*^R^-FRT	
KT148	pKN11/KT32	This study
KT152	pKN11/KT43	This study
KT153	pKN11/KT105	This study
KT154	pKN11/KT112	This study
KT159	MG1655 except Δ*argR*::FRT	This study
	Δ*potE*::FRT Δ*speD*::FRT Δ*speG*::FRT	
	Δ*argA*::FRT-*kan*^R^-FRT	
KT160	pKN11/SH2204	This study
KT161	pKN11/SH2206	This study
KT162	pKN11/KT159	This study
KT207	pSH1820/FS115	This study
KT208	pKT199/FS115	This study
KT209	pKT200/FS115	This study
KT210	pKT201/FS115	This study
SH2201	MG1655 except Δ*argR*::FRT	This study
	Δ*patA*::FRT Δ*potE*::FRT Δ*speD*::FRT	
	Δ*speG*::FRT Δ*argA*::FRT	
	Δ*puuPA*::FRT Δ*yifE*::FRT-*kan*^R^-FRT	
SH2203	*yifE*^Q100TAG^ Δ*ybbA*::FRT *zie*-*296*::Tn*10* This study	
SH2204	MG1655 except Δ*argR*::FRT	This study
	Δ*patA*::FRT Δ*potE*::FRT Δ*speD*::FRT	
	Δ*speG*::FRT Δ*argA*::FRT	
	Δ*puuPA*::FRT *yifE*^Q100TAG^ *zie*-*296*::Tn*10*	
SH2206	SH2204 except *potFGHI*::FRT-*kan*^R^-FRT	This study

### Plasmid construction

The plasmids used in this study are shown in Table 2 and the primers used to construct the plasmids are listed in Additional file 1: Table S2. The Wizard Plus SV Minipreps DNA Purification System (Promega; Madison, WI) was used to extract plasmid DNA from cells. PCR amplification was performed using KOD-plus DNA polymerase (Toyobo; Osaka, Japan). The QuikChange method (Stratagene; San Diego, CA) was used to generate *argA*^ATG Y19^ of pSH1733 using KOD-plus DNA polymerase. There is a 137-base pair space between the *speA* and *speB* genes in the genome of MG1655. Therefore, the wild-type *speAB* genes may not be an operon. The *speA* and *speB* genes on the plasmids used in this study are different from the wild-type genes on the genome. They are designed to have only a 21-base pair space with a Shine Dalgarno (SD) sequence between them, i.e., they were designed to be an operon. Consequently, the transcription of *speA* will also pass through *speB*. The *speC* gene was amplified using genomic DNA of MG1655 as a template and primers with *Eco*RI and *Sac*I recognition sites. After the amplified fragment was cleaved with these two enzymes, it was inserted between the *Eco*RI and *Sac*I sites of pQE-80L vector.

**Table 2:** Plasmids used in this study

Strains	Genotype	Source or reference
pCP20	pSC101 replicon (Ts) *bla*^+^ *cat*^+^	Datsenko and Wanner 2000
	Flp(*γ*Rp) d857	
pFS29	pQE-80L except T5p *lacO_lacO_speC*^+^	This study
pKD13	*oriR*γ *bla*^+^ FRT-*kan*^R^-FRT	Datsenko and Wanner 2000
pKN11	pQE-80L except T5p *lacO_lacO*	This study
	*_speA_speB_argA*^ATG Y19C^	
pKN18	pQE-80L except T5p *lacO_lacO*	This study
	*_speA_speB_argA*^ATG Y19C^ T5p *lacO_*	
	*lacO_speC*	
pKT199	pQE-80L except	This study
	*lacI*^q1^p*_lacO_speA_speB_argA*^GTG Y19C^	
	*lacI*	
pKT200	pQE-80L except	This study
	*lacI*^q1^p*_lacO_speA_speB_argA*^ATG Y19^	
	*lacI*	
pKT201	pQE-80L except	This study
	*lacI*^q1^p*_lacO_speA_speB_argA*^GTG Y19^	
	*lacI*	
pQE-80L	ColE1 replicon *bla*^+^ *lacI*^q^ T5p	Qiagen
	*lacO*_*lacO*-(His)6	
pSH1733	pQE-80L except *argA*^ATG Y19C^	This study
pSH1734	pQE-80L except T5p *lacO_lacO_speC*^+^	This study
	_ *argA*^ATG Y19C^	
pSH1807	pQE-80L except	This study
	*lacI*^q^p*_lacO_speA_speB_argA*^ATG Y19C^	
	*lacI*	
pSH1820	pQE-80L except	This study
	*lacI*^q1^p*_lacO_speA_speB_argA*^ATG Y19C^	
	*lacI*	
pSH1823	pQE-80L except	This study
	*lacI*p*_lacO_speA_speB_argA*^ATG Y19C^	
	*lacI*	

### Medium and culture conditions for putrescine production

Pre-culture was carried out in 10 mL of LB medium in a 100-mL Erlenmeyer flask for 16 h at 37 °C with a reciprocal shaking at 120 rpm. After 16 h, the pre-culture was transferred to 10 mL of LB or terrific broth medium to adjust the initial density of the cells to an OD_600_ of 0.05. The cultures were incubated at 37 °C with a reciprocal shaking at 120 rpm until the turbidity OD_600_ reached 0.4. Then, IPTG was added to the main culture as needed. When ampicillin was required, 100 μg/mL of ampicillin was added to the media.

### Sample preparation

The culture was collected at the indicated time and used to measure the turbidity at OD_600_. The collected cultured was centrifuged at 18,000 × g at 4 °C for 5 min. The supernatants were collected, and 25 μL of 100% (w/v) trichloroacetic acid was mixed with 250 μL of the three supernatants. Samples were filtrated through a Millex-LH Syringe Driven Filter Unit (Millipore; Billerica, MA).

### Measurement of putrescine concentrations

The concentration of putrescine was measured using an LC-20 HPLC device (Shimadzu; Kyoto, Japan) equipped with a TSKgel Polyaminepak (Tosoh; Tokyo, Japan), as described previously (Kurihara et al. 2008). The temperature of the column was set at 50 °C and only one mobile phase solution was used. The flow rate of the mobile phase solution was 0.5 mL/min and that of the detection reagent was 0.42 mL/min. The peak of putrescine was detected at 6 min under this analytical condition. Putrescine was purchased from Nacalai Tesque (Kyoto, Japan). The amounts of polyamines are presented as the average of three independent cultures with standard deviations.

### Data analysis

All data were analyzed using SPSS Statistics 25 (IBM; Armonk, NY). The one-way analysis of variance (ANOVA) was used to determine significant differences.

## Results

### Effects of the initiation codon and Y19C substitution of ArgA

**Fig. 2:**
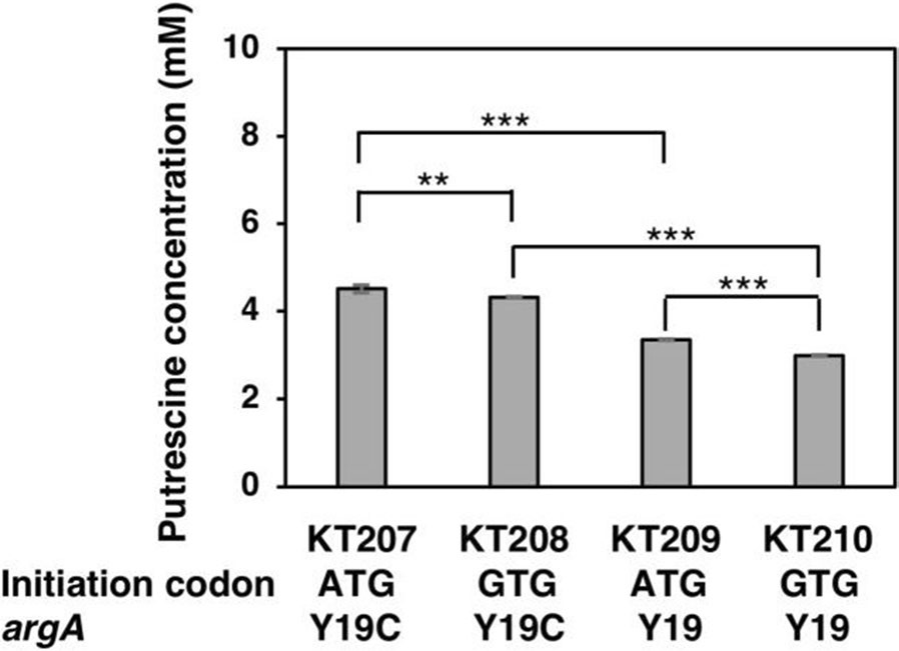
Base substitution on *argA* increased putrescine production. Putrescine concentration in the supernatant of strains harboring plasmids with different mutant *argA*: KT207 (*argA*^ATG Y19C^), KT208 (*argA*^GTG Y19C^), KT209 (*argA*^ATG Y19^), and KT210 (*argA*^GTG Y19^). The strains were grown in LB medium supplemented with 100 μg/mL of ampicillin at 37 °C for 48 h with reciprocal shaking at 120 rpm. When the OD_600_ reached 0.4, 0.02 mM IPTG was added. **, p < 0.01; ***, p < 0.001. Data shown are averages ± standard deviations, and culture experiments were performed in triplicate

The wild-type strain, MG1655 excretes only 0.18 mM of putrescine in LB medium (data not shown). As arginine is a key substrate of putrescine synthesis through the ADC pathway, to increase putrescine production, we constructed plasmids with the ArgA desensitized to the feedback inhibition by arginine. In addition, the initiation codon of native ArgA is GTG, which is inefficient in translation initiation. Therefore, we generated a plasmid containing the *argA* gene with the effective ATG codon. Moreover, *speA* and *speB* genes were introduced under the *lacI*^q1^ promoter along with the *argA* gene in order to improve putrescine production. To compare the effects of both amino acid and initiation codon substitution, pSH1820 (*argA*^ATG Y19C^), pKT199 (*argA*^GTG Y19C^), pKT200 (*argA*^ATG Y19^), and pKT201 (*argA*^GTG Y19^) were constructed, and strain FS115 was transformed to obtain KT207, KT208, KT209, and KT210, respectively. The strains were grown in LB medium with ampicillin and incubated at 37 °C. The result is illustrated in Fig. 2. KT207 excreted the highest concentration of putrescine into the medium, whereas strain KT210 excreted the lowest amount of putrescine. To compare the effects of the initiation codon, the strains were separated into two groups: GTG codon (KT208 and KT210) and ATG codon (KT207 and KT209). ATG codon exhibited a higher concentration of putrescine than the original initiation codon, GTG. In addition, Y19C substitution in strains KT207 and KT208 resulted in notably higher putrescine production than strains containing the native amino acid residue Y19 (KT209 and KT210).

### Effect of SpeA, SpeB, and SpeC on putrescine production

**Fig. 3:**
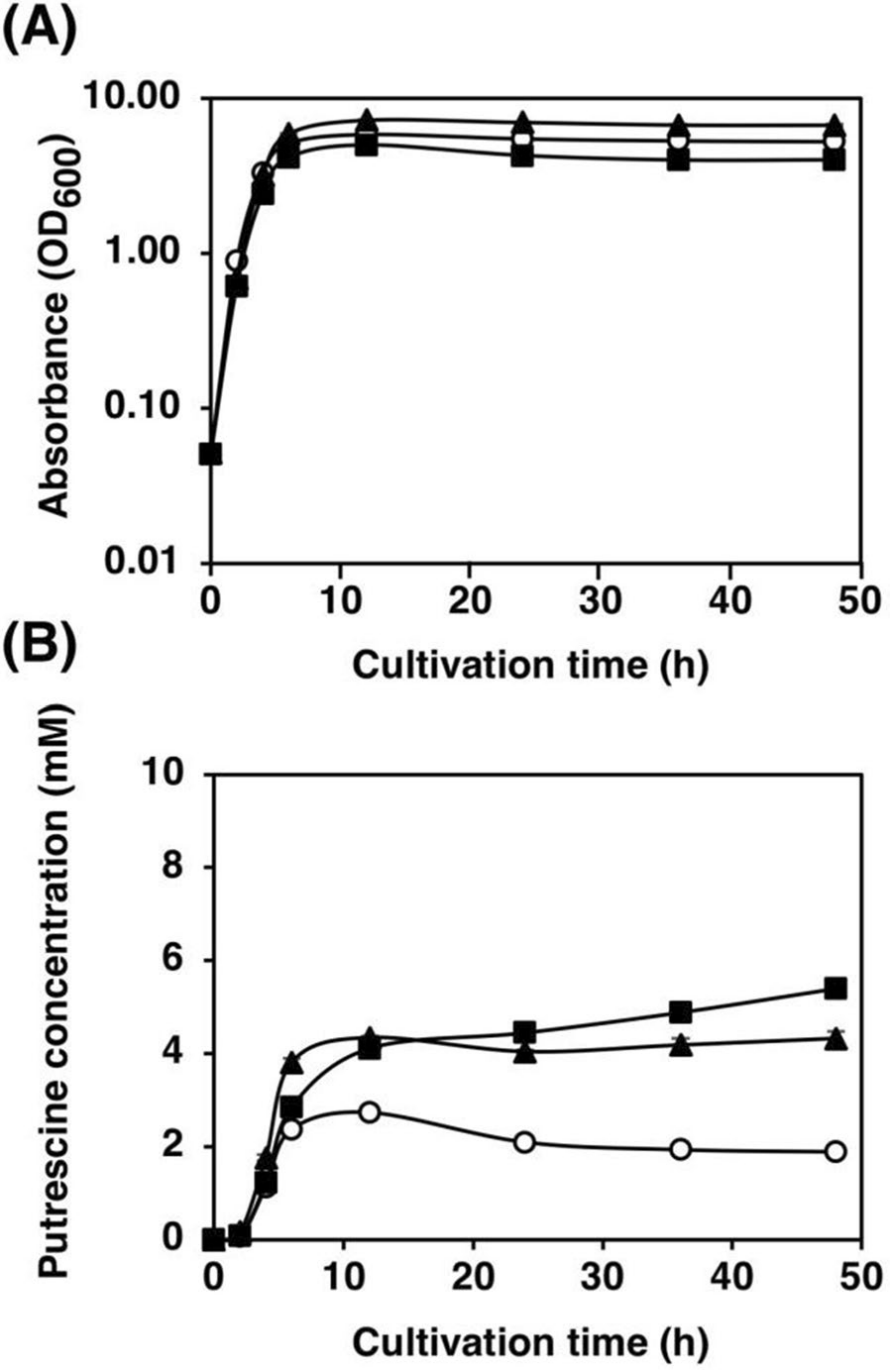
Effect of SpeA, SpeB, and SpeC on putrescine production. **A** Bacterial cell growth and **B** putrescine concentration in the supernatant of strains FS123 (*speC*^+^, open circle), KN20 (*speABC*^+^, closed triangle), and KN24 (*speAB*^+^, closed square) grown in LB medium supplemented with 100 μg/mL of ampicillin. When the OD_600_ reached 0.4, 0.02 mM IPTG was added. Data shown are averages ± standard deviations, and culture experiments were performed in triplicate

There are ADC and ODC pathways for putrescine synthesis in *E. coli*. Whether the over-expression of ADC pathway only or of both ADC and ODC pathways is appropriate for the over-production of putrescine was not sure. To address this issue, we constructed a plasmid that has *argA*^ATG Y19C^ together with only *speC* gene under T5 promoter (pFS29) and compare with another two plasmids which have *argA*^ATG Y19C^ with only *speAB* genes (pKN11) and *argA*^ATG Y19C^ with *speAB* and *speC* genes (pKN18). The plasmids were transformed into MG1655 as a host strain, resulting in FS123, KN24, and KN20, respectively. As depicted in Fig. 3A, the cell growth profiles of the above three strains were not different. However, the putrescine production of KN24, which has only *speAB* genes on a plasmid with *argA*^ATG Y19C^ under T5 promoter, exhibited the highest putrescine content among these three strains (Fig. 3B). Therefore, the plasmid pKN11, which contains only *speAB* genes with *argA*^ATG Y19C^, was selected to use in further experiments.

### Effects of wild-type ArgA on ArgA^ATG Y19C^

**Fig. 4:**
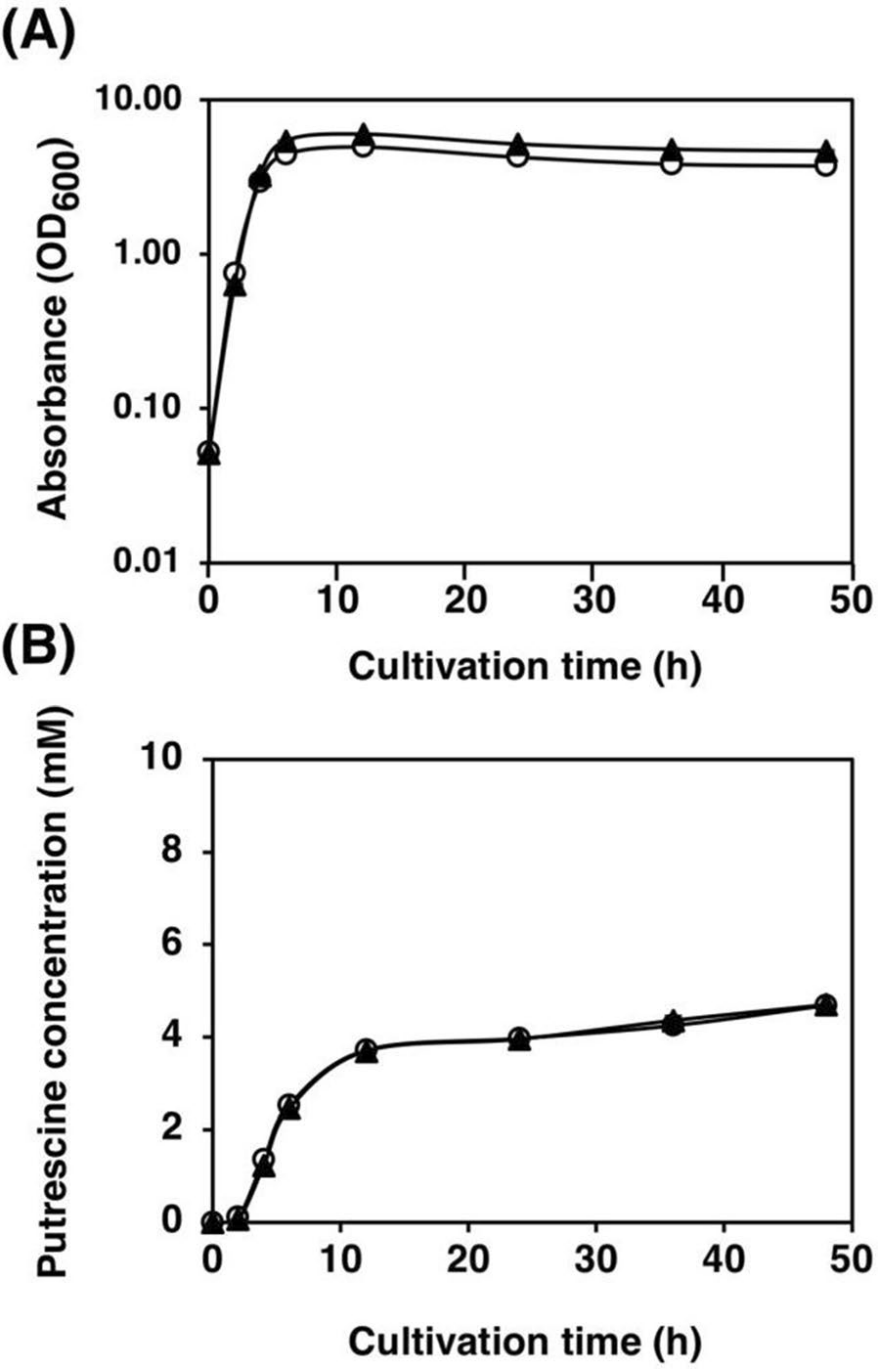
Effects of the wild-type ArgA expressed from the genome on putrescine production. **A** Bacterial cell growth and **B** putrescine concentration in the supernatant of strains KT39 (Δ*argA*, open circle) and KT207 (*argA*^+^, closed triangle) grown in LB medium supplemented with 100 μg/mL of ampicillin. When the OD_600_ reached 0.4, 0.02 mM IPTG was added. Data shown are averages ± standard deviations, and culture experiments were performed in triplicate

ArgA forms a homohexamer to be an active enzyme. When genomic *argA* is wild-type, it may form a mixed hexamer of ArgA^wt^ and ArgA^Y19C^. ArgA^wt^ may be dominant to ArgA^Y19C^ in the desensitization of arginine. To compare the effects of wild-type ArgA, the strains with and without *argA*, FS115 (*argA*^+^) and KT32 (Δ*argA*), respectively, were constructed. The plasmid carrying *speAB* and *argA* (*argA*^ATG Y19C^) encoding desensitized ArgA^ATG Y19C^ under the regulation of the *lacI*^*q1*^ promoter and *lac* operator was constructed (pSH1820) and used to transform the above-mentioned strains (FS115 and KT32), resulting in KT207 and KT39, respectively. To evaluate the effects of the coexistence of wild-type ArgA and desensitized ArgA^ATG Y19C^, KT207 and KT39 were used. As depicted in Fig. 4, the growth profile and putrescine production did not significantly differ between KT207 and KT39.

### Effects of promoter strength on putrescine production

**Fig. 5:**
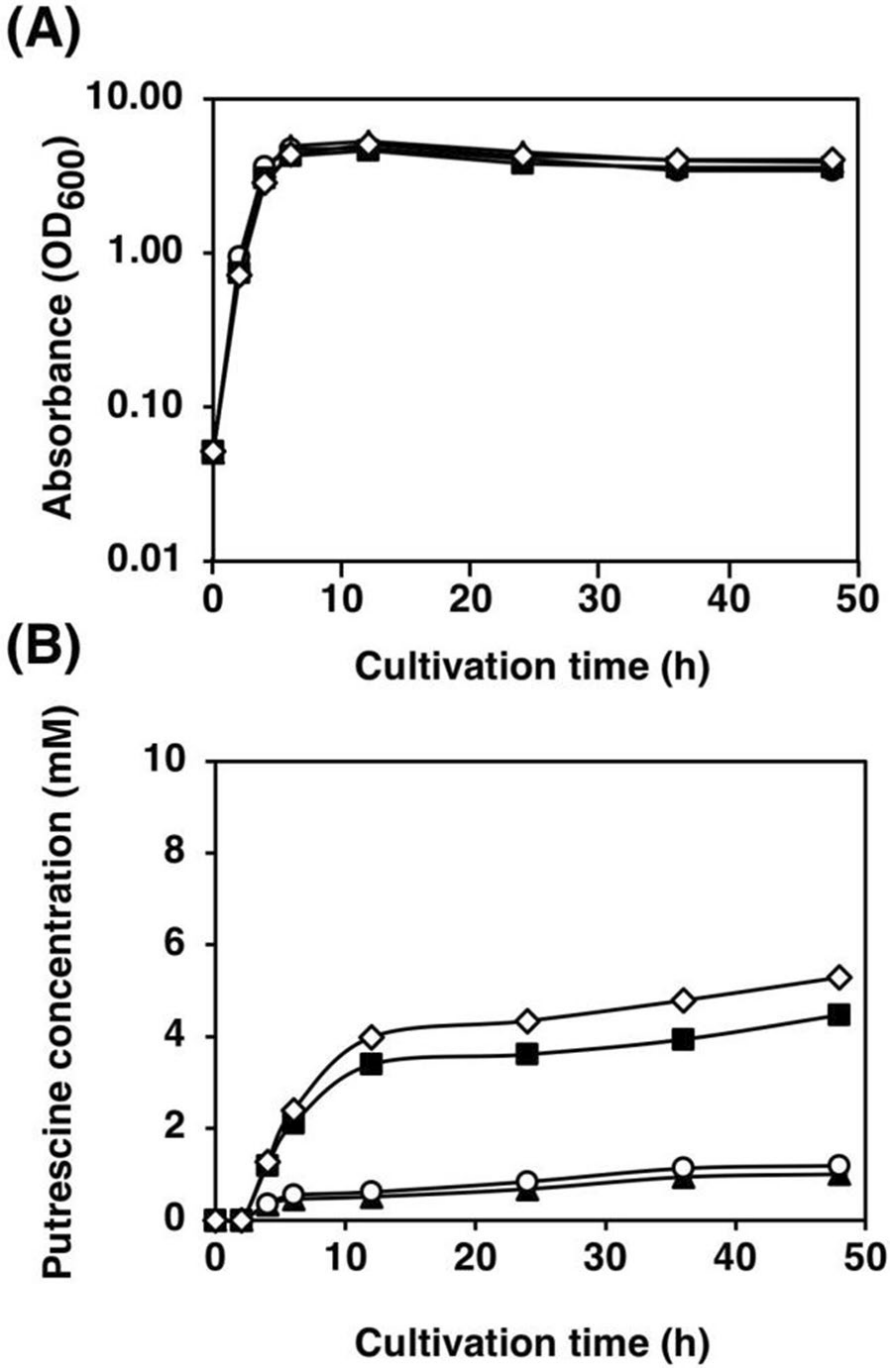
Effects of promoters of *speAB* and desensitized *argA* on putrescine production. **A** Bacterial cell growth and **B** putrescine concentration in the supernatant of strains KT37 (*lacI* promoter, closed triangle), KT38 (*lacI*^q^ promoter, open circle), KT39 (*lacI*^q1^ promoter, closed square), and KT148 (T5 promoter, open diamond) grown in LB medium supplemented with 100 μg/mL of ampicillin. When the OD_600_ reached 0.4, 0.02 mM IPTG was added. Data shown are averages ± standard deviations, and culture experiments were performed in triplicate

To promote the sequential reactions from arginine to putrescine, the promoter for *speAB*_*argA*^ATG Y19C^ was evaluated. The concentrations of IPTG for inducible *lacI*, *lacI*^q^, *lacI*^q1^, and T5 promoters were compared. The *lacI* promoter of pSH1823 was replaced by *lacI*^q^ and *lacI*^q1^promoters, resulting in pSH1807 and pSH1820, respectively. The plasmid (pKN11) carrying the *speAB*_*argA*^ATG Y19C^ under the T5 promoter and two *lac* operators was constructed. Strain KT32 was transformed with each plasmid to obtain KT37 (*lacI*p), KT38 (*lacI*^q^p), KT39 (*lacI*^q1^p), and KT148 (T5p). These strains were grown in LB medium with the addition of 0.02 mM IPTG. The putrescine production of KT38 (*lacI*^q^p) was not different from that of KT37 (*lacI*p). On the other hand, strains KT39 (*lacI*^q1^p), and KT148 (T5p) produced 349% and 431% more putrescine than strain KT37 (*lacI*p), respectively (Fig. 5B). Based on these results, KT148, carrying *speAB* and desensitized *argA* under the T5 promoter and two *lac* operators, was used for further study. To optimize the IPTG concentration, it was varied from 0 to 0.20 mM. Among these concentrations, 0.03 mM IPTG resulted in the highest putrescine production by KT148 in LB medium (data not shown).

### Disruption of genomic *puuPA* increases putrescine production

**Fig. 6:**
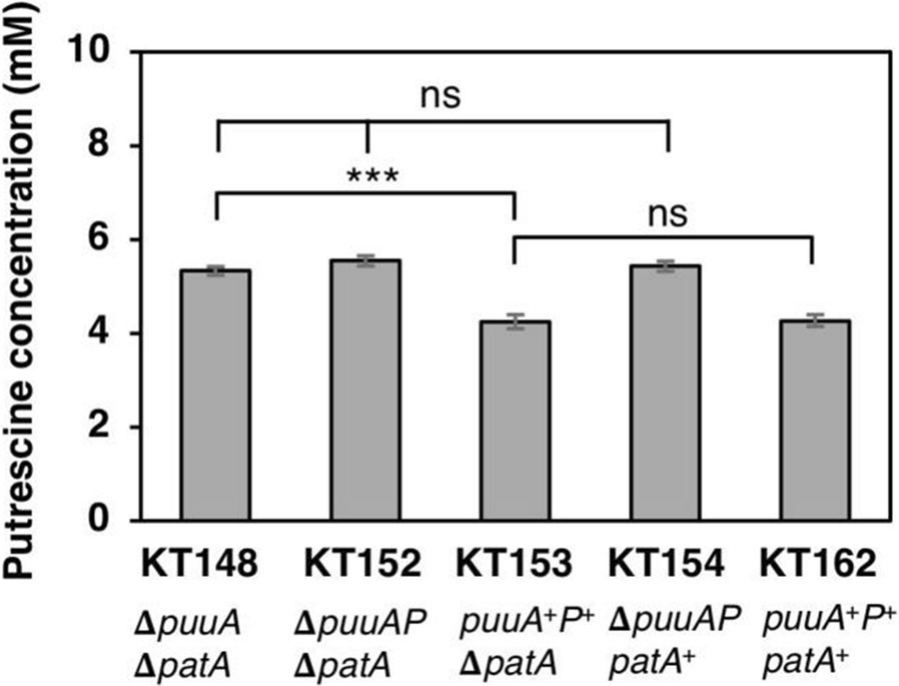
Effects of *patA*, *puuA*, and *puuP* genes on the putrescine production. The amount of putrescine in the culture supernatant of the strains KT148, KT152, KT153, KT154, and KT162 grown in LB medium supplemented with 100 μg/mL of ampicillin for 48 h. When the OD_600_ reached 0.4, 0.03 mM IPTG was added. ***, p < 0.001; ns, not significantly different. Data shown are averages ± standard deviations, and culture experiments were performed in triplicate

To improve the accumulation of extracellular putrescine, the importer and degradation enzymes of putrescine should be eliminated. The host strains KT32 (Δ*puuA* Δ*patA*), KT43 (Δ*puuAP* Δ*patA)*, KT105 (*puuP*^+^*A*^+^ Δ*patA)*, KT112 (Δ*puuAP patA*^+^), and KT159 (*puuP*^+^*A*^+^ *patA*^+^) were constructed to assess the effects of *patA*, *puuA*, and *puuP* on putrescine production. The above strains were transformed by the plasmid pKN11 (T5p_*speAB*_*argA*^ATG Y19C^), leading to strains KT148, KT152, KT153, KT154, and KT162. Among all strains, *puuP*^+^*A*^+^ strains (KT153 and KT162) produced a lower amount of putrescine than the Δ*puuA* (KT148) and Δ*puuAP* (KT152 and KT154) strains (Fig. 6B). However, there were no significant differences in the concentration of extracellular putrescine between KT153 (Δ*patA*) and KT162 (*patA*^+^) strains.

### Effects of the medium on putrescine production

**Fig. 7:**
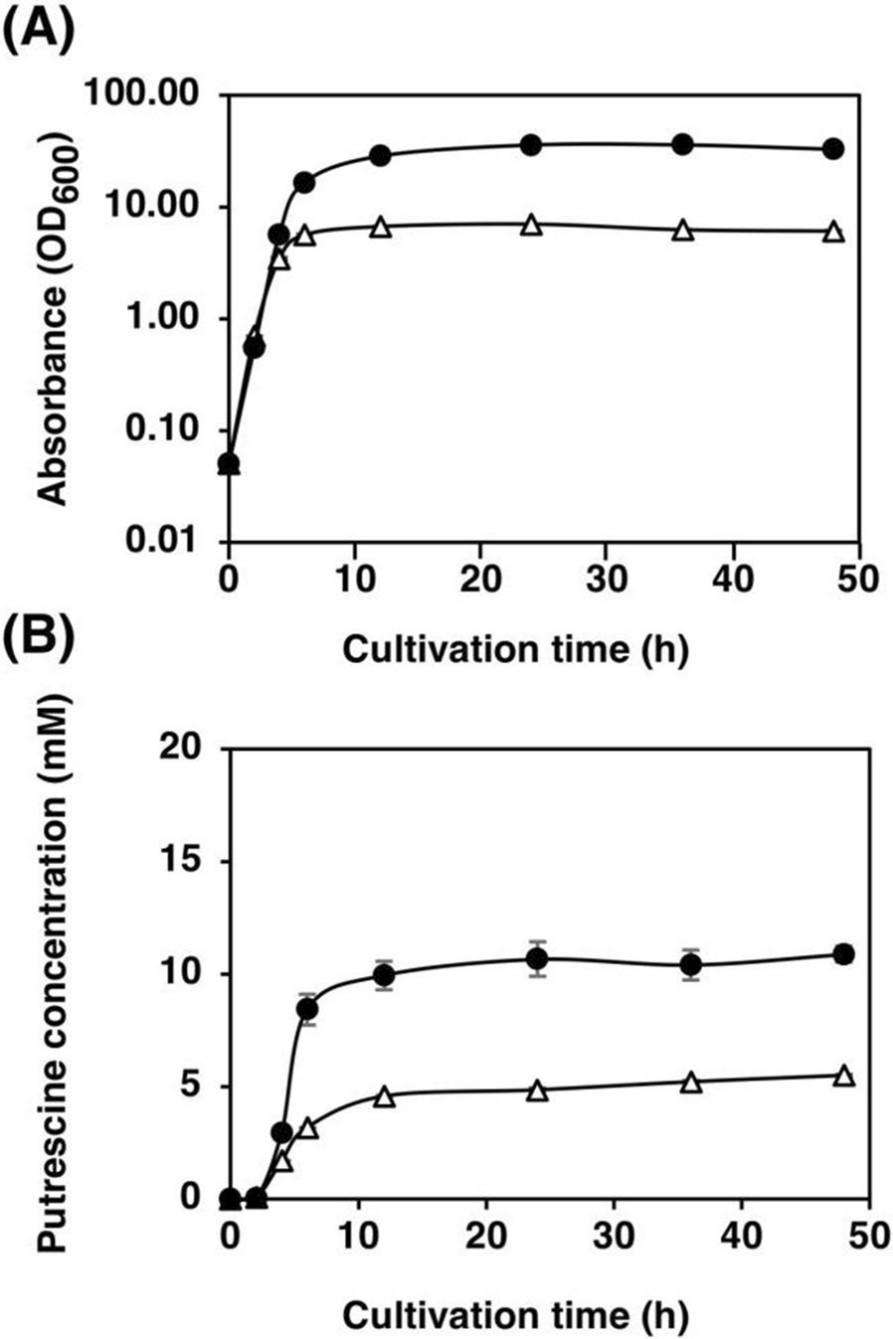
Effects of culture media on putrescine production by strain KT152. **A** Bacterial cell growth and **B** putrescine concentration in the supernatant of the strain KT152, whose putrescine utilization pathway (Δ*puuAP* Δ*patA*) was disrupted. Open triangles represent the strain KT152 grown in the LB medium supplemented with 100 μg/mL of ampicillin; closed circles represent the strain KT152 grown in terrific broth supplemented with 100 μg/mL of ampicillin. When the OD_600_ reached 0.4, 0.03 mM IPTG was added. Data shown are averages ± standard deviations, and culture experiments were performed in triplicate

The amount of putrescine production was compared using LB medium and terrific broth to optimize the medium for putrescine production. Strain KT152 grew and produced putrescine more in terrific broth than in LB (Fig. 7). At 48 h of incubation, strain KT152 excreted 10.9 mM of extracellular putrescine in terrific broth, which was about twice as much as that in LB (Fig. 7B). Moreover, the IPTG concentration was optimized for cultivation in terrific broth. The concentration of IPTG was varied from 0 to 0.20 mM. The optimal concentration of IPTG for KT152 cultured in terrific broth was 0.02 mM (data not shown).

### Effects of putrescine transporter PotFGHI and mutant YifE^Q100TAG^

**Fig. 8:**
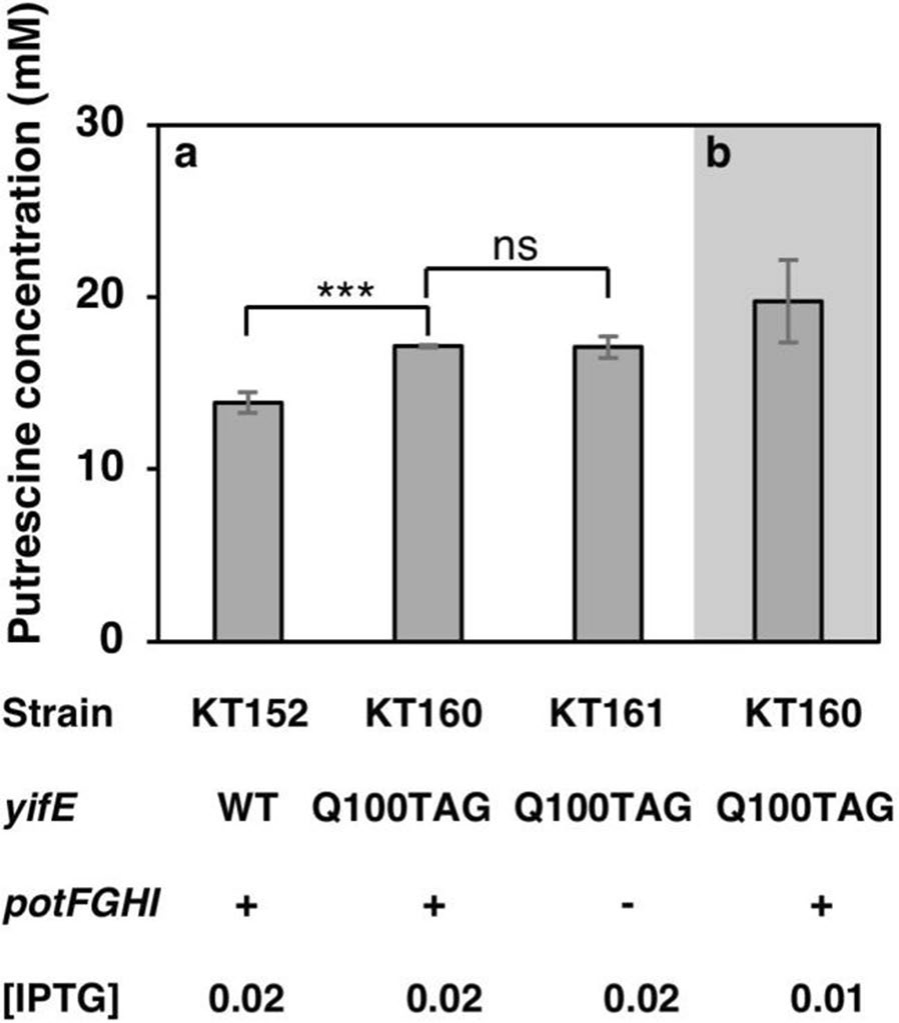
Effects of the *yifE* mutant and putrescine transporter PotFGHI on putrescine production. **a** The putrescine concentration in the supernatant of the culture media of strains KT152, KT160, and KT161 grown in terrific broth supplemented with 100 μg/mL of ampicillin for 48 h. When the OD_600_ reached 0.4, 0.02 mM IPTG was added. **b** The putrescine concentration in the supernatant of the culture media of strains KT160 grown in terrific broth supplemented with 100 μg/mL of ampicillin. When the OD_600_ reached 0.4, 0.01 mM IPTG was added. * p < 0.001; ns, not significantly different. *yifE*: WT, *yifE*^wt^; Q100TAG, *yifE*^Q100TAG^ gene. *potFGHI*: + , presence of *potFGHI* operon; -, absence of *potFGHI* operon. [IPTG]: 0.01 and 0.02 is the optimum concentration of IPTG in terms of mM. Data shown are averages ± standard deviations, and culture experiments were performed in triplicate

To clarify whether the disruption of the PotFGHI transporter and the mutation on YifE improved putrescine production, strain SH2204 was transformed with *yifE*::FRT-*kan*^R^-FRT of SH2201 and the *yifE*^Q100TAG^ was replaced using *zie-296*::Tn*10* as a co-transduction marker by P1*vir* phage grown on SH2203. The *potFGHI* operon of the strain SH2204 (*yifE*^Q100TAG^) was disrupted by P1*vir* transduction to obtain strain SH2206 (*yifE*^Q100TAG^ Δ*potFGHI*). Strains KT43, SH2204, and SH2206 were transformed with pKN11 (T5p_*speAB*_*argA*^ATG Y19C^), and strains KT152, KT160, and KT161 were obtained. Putrescine production by these strains in a terrific broth with the addition of 0.02 mM IPTG is compared in Fig. 8a. Samples were taken at 0, 2,4, 6, 12, 24, 36, and 48 h of incubation. Among these sampling times, the highest concentration of putrescine was observed at 48 h (data not shown). There was no significant difference in putrescine production between strains KT160 and KT161. However, the amount of extracellular putrescine excreted from both KT160 (*yifE*^Q100TAG^ *potFGHI*^+^) and KT161 (*yifE*^Q100TAG^ Δ*potFGHI*) was 23% higher than that by KT152 (*yifE*^+^ *potFGHI*^+^) (Fig. 8a). In addition, the IPTG concentration was optimized from 0 to 0.2 mM for the cultivation of KT160 in terrific broth. The optimal concentration of IPTG for KT160 cultured in terrific broth was 0.01 mM and the strain produced 19.8 mM of putrescine (Fig. 8b).

## Discussion

*Efficient initiation codon of argA*^*Y19C*^* improved the putrescine production-* Arginine is a key substrate of putrescine synthesis through the ADC pathway. The biosynthetic pathway of arginine initiates with glutamate to *N*-acetylglutamate by *N*-acetylglutamate synthase (ArgA), which is encoded by *argA*. However, not only is the transcription of the *argA* gene repressed by ArgR, but the activity of ArgA is also inhibited by the final product arginine (Lu 2006; Sun et al. 2015; Xu and Zhang 2019a, b). To produce putrescine from glutamate, the repression of *argA* by ArgR and the feedback inhibition of ArgA by arginine are of concern. Previously, Rajagopal et al. (1998) constructed plasmids with a point mutation in the *argA* gene. Among all point mutations, the strain with the Y19C mutation, which is an amino acid substitution of tyrosine with cysteine at the 19th codon, was able to produce the highest amount of arginine. According to the review of Igarashi and Kashiwagi (2010), the addition of putrescine stimulates the translation of polyamine modulons. As ArgA is a polyamine modulon (Igarashi et al. 2015), its translation can be stimulated by the increase in putrescine. The effects of the substitution of the GTG codon with the ATG codon was evaluated, but the substitution of GTG with ATG was not so effective as expected (Fig. 2). This may be because the plasmid we constructed has only 8 base pairs between the SD sequence and the ATG initiation codon, and it lacks the characteristics of a polyamine modulon. However, desensitization of ArgA by amino acid substitution at 19 positions from tyrosine to cysteine from the plasmid increased putrescine production (Fig. 2). And the strain with *argA*^ATG Y19C^ had the highest amount of putrescine.

*The effects of wild-type ArgA on desensitized ArgA-* To confirm whether ArgA^wt^ or ArgA^ATG Y19C^ is dominant, we compared the putrescine production of KT39 (*argA*^ATG Y19C^/Δ*argA*) and KT207 (*argA*^ATG Y19C^/*argA*^+^) strains. The growth and putrescine production of both KT39 and KT207 were not different. Thus, the presence of wild-type ArgA expressed from the genome does not affect the growth or putrescine production (Fig. 4).

*The strong promoter T5*p *increases putrescine production-* According to the report of Buch and Boyle (1985), a significant proportion of SpeA associates with the cell envelope. A recent paper reported that some of SpeA is in the periplasm and the other is in the cytoplasm (Meydan et al. 2019). Overexpression of membrane-associated or excreted proteins can affect cell health. Therefore, in this study, the promoter strength required for overexpression was of concern. The *lacI*, *lacI*^q^, *lacI*^q1^, and T5 promoters were compared. The strain carrying the *lacI* promoter produced the lowest concentration of extracellular putrescine, whereas the strain carrying the T5 promoter produced the highest. Due to its affinity for RNA polymerase, the *lacI* promoter is the poorest (Muller-Hill et al. 1968). Therefore, the expression level is low. A point mutation at the − 35 region of the *lacI*^q^ promoter led to tenfold higher expression and the *lacI*^q1^ promoter resulted in 170-fold higher expression (Calos 1978; Glascock and Weickert 1998; Penumetcha et al. 2010). Moreover, there are two *lac* operators in this T5 promoter with a strong affinity to *E. coli* RNA polymerase (von Gabain and Bujard 1977, 1979; Shibui et al. 1988), but no study has directly compared their affinity for RNA polymerase. As depicted in Fig. 5B, the putrescine production of KT148 (T5p) was higher than that of KT39 (*lacI*^q1^p).

*Effects of puuPA disruption on putrescine production-* Two catabolic pathways of putrescine have been reported. PatA and PuuA are the first enzymes of the aminotransferase pathway and putrescine utilization pathway (the Puu pathway), respectively. PatA transfers one of the amino groups of putrescine to α-ketoglutarate (Shaibe et al. 1985; Schneider and Reitzer 2012). Another enzyme that catabolizes putrescine is PuuA, which γ-glutamylates the amino group of putrescine to generate γ-glutamylputrescine. The proteins involved in the Puu pathway are encoded in a gene cluster. The importer of this Puu pathway is PuuP, which is encoded in this gene cluster. After PuuP transports putrescine into the cell, the degradation step begins with PuuA (Kurihara et al. 2009b). There is no report to compare the reaction velocity or the expression level between the *puuA* and *patA* yet. So, it is difficult to specify which enzymes are mainly work. However, according to our previous study, the deletion of the *puuA* gene caused a severe effect on putrescine utilization and Δ*puuA* strain could not grow using putrescine as a nitrogen source or a carbon source, while the deletion of the *patA* gene did not affect this issue (Kurihara et al. 2008). The result in this study is agreeable with the previous study that the presence of *puuA* showed the degradation of putrescine while *patA* is not. In addition, under the condition in which putrescine is present, PuuP is required as a putrescine transporter to use putrescine as an energy source (Kurihara et al. 2009b; Terui et al. 2014). As PuuP may also have its own promoter, we constructed KT148 (∆*puuA*) and KT152 (∆*puuAP*) to investigate the relationship between these *puuAP* genes. As shown in Fig. 6, *puuAP* may be an operon because ∆*puuAP* had the same result as ∆*puuA*. The deletion of *puuA* increased the extracellular accumulation of putrescine. In summary, PatA does not affect putrescine production, but the deletion of PuuA increases the amount of putrescine.

*Terrific broth is a better medium for putrescine production-* LB medium is widely used as a standard medium for a broad group of bacteria (Sezonov et al. 2007; Suzuki et al., 2019). However, the growth of *E. coli* is not high in the LB medium, whereas it is markedly high in terrific broth (Losen et al. 2004; Lessard 2013). Terrific broth contains 5.04 g/L of glycerol, while LB medium does not. *E. coli* can utilize glycerol as a carbon source for growth and putrescine production. Besides much higher concentration of yeast extracts, this is one of the reasons why the growth and putrescine production in terrific broth is better than those in LB medium (Fig. 7).

*Mutation of YifE increases putrescine*- During we attempted to find a putrescine exporter in *E. coli* K-12, we found that the extracellular putrescine concentration of strain JW0484 with Δ*ybbA*::FRT-*kan*^R^-FRT was approximately double that of its parental strain, ME9062 (Additional file 1: Fig. S1). The Δ*ybbA*::FRT-*kan*^R^-FRT was transduced to strain MG1655 to confirm the increase of putrescine production. However, the extracellular putrescine concentration of the MG1655 with Δ*ybbA*::FRT-*kan*^R^-FRT was not different from that of MG1655. We predicted that the second mutation in strain JW0484 increased the extracellular putrescine concentration. Then, its genome sequence was performed to evaluate this prediction. The result of genome sequencing showed several mutations in strain JW0484, which were not reported in strain ME9062. Additional mutations were found in *intA*, *fbaA*, *dgoR*, *yifE*, and *lexA* in the genome of strain JW0484. Further experiments revealed that mutation of the *yifE* gene increases the extracellular putrescine concentration (Additional file 1: Fig. S2). As shown in Fig. 8a, the YifE mutant was also effective in our putrescine over-producing strain (KT160). RT-PCR revealed that the *yifE*^Q100TAG^ mutation increases the transcription of *hdfR* and *gltB* genes.

Pistocchi et al. (1993) reported that the PotFGHI transporter uptakes putrescine and accumulates it in the cell. Therefore, it is conceivable that PotFGHI reduces extracellular putrescine. We compared the strains with and without the PotFGHI transporter, but there was no difference in the concentration of putrescine between *potFGHI*^+^ and Δ*potFGHI* strains. Thus, the PotFGHI transporter does not affect the amount of extracellular putrescine. Terui et al. (2014) reported that PotFGHI functions as a major transporter when putrescine is absent in the medium. However, the PotFGHI transporter can be inhibited by putrescine through a feedback loop. In our experimental condition, putrescine was synthesized and exported to the medium; therefore, PotFGHI may have been inhibited by putrescine. We achieved 19.8 mM of putrescine production from KT160 in terrific broth. When compare in terms of the product yield, Qian et al. (2009) reported a yield of putrescine at 30.3 g/M glucose, while our putrescine production is the 31.8 g/M glycerol. However, as putrescine is an ionic compound, its production altered the pH of the medium during cultivation. As we do not have a bioreactor device, we were unable to assess continuous feeding with pH adjustment.

## Supplementary Information

**Additional file 1: Table S1**. Primers used for strain construction. **Table S2**. Primers used for plasmid construction. **Table S3**. Primers used for Realtime PCR. **Fig. S1**. Comparison of extracellular putrescine concentration per OD_600_ among strains of the Keio collection. M9 glucose medium (60 mL in 100-mL Erlenmeyer flask with stirrer bar) was inoculated with the pre-culture at the initial OD_600_ of 0.06. The flasks were set in the 7-L rectangular anaerobic jar with 2 AnaeroPack-Anaero sachets (Mitsubishi Gas Chemical; Tokyo, Japan) to form an anaerobic environment and the jar was set on the stirrer in the microbiological incubator. The culture medium was stirred at 150 rpm and kept at 37°C. After 10 h of incubation, the OD_600_ of the culture was measured and 0.4 mL of the culture was centrifuged. Thirty μL of 100% (w/v) TCA was mixed with 0.3 mL of the supernatant, filtrated through the membrane filter, and then subjected to HPLC analysis as described in the main text. Asterisks indicate the genes with significant differences in transcription levels. **Fig. S2**. Q100TAG mutation in the *yifE* gene promotes putrescine production. Extracellular concentration of putrescine of AI32 (pQE-80L/MG1655, open diamond) and AI33 (pQE-80L::*yifE*^Q100TAG^/MG1655, closed square) cultured at 37°C in minimal M9 supplemented with 0.2% glucose. 100 μg/mL of ampicillin was added to maintain the plasmids. When the OD_600_ reached 0.5, the 0.5 mM IPTG was added. **Fig. S3**. The presence of *hdfR* had a slight, if any, effect on the increase in extracellular putrescine concentration by YifE^Q100TAG^. (A) Cell growth and (B) extracellular concentration of putrescine of AI32 (pQE-80L/MG1655, open circle), AI33 (pQE-80L::*yifE*^Q100TAG^/MG1655, closed triangle), and KT218 (pQE-80L::*yifE*^Q100TAG^/ MG1655 but Δ*hdfR*::FRT-*kan*^R^-FRT, closed square) cultured at 37°C in LB supplemented with 100 μg/mL of ampicillin. When the OD_600_ reached 0.4, 0.02 mM IPTG was added.

## Data Availability

Some data are not shown in the text and supplement. However, they are available from the authors upon reasonable request. The strains and plasmids used in this work can be shared by exchanging MTA.
