# Supplementary material for: Improvement of putrescine production through the arginine decarboxylase pathway in Escherichia coli K-12
Source: AMB Express. 2021 Dec 15;11:168. doi: 10.1186/s13568-021-01330-5 (PMC8674398; doi:10.1186/s13568-021-01330-5)
Supplement: Supplementary file 1 — Additional file 1: Table S1. Primers used for strain construction. Table S2. Primers used for plasmid construction. Table S3. Primers used for Realtime PCR. Fig. S1. Comparison of extracellular putrescine concentration per OD600 among strains of the Keio collection. M9 glucose medium (60 mL in 100-mL Erlenmeyer flask with stirrer bar) was inoculated with the pre-culture at the initial OD600 of 0.06. The flasks were set in the 7-L rectangular anaerobic jar with 2 AnaeroPack-Anaero sachets (Mitsubishi Gas Chemical; Tokyo, Japan) to form an anaerobic environment and the jar was set on the stirrer in the microbiological incubator. The culture medium was stirred at 150 rpm and kept at 37°C. After 10 h of incubation, the OD600 of the culture was measured and 0.4 mL of the culture was centrifuged. Thirty μL of 100% (w/v) TCA was mixed with 0.3 mL of the supernatant, filtrated through the membrane filter, and then subjected to HPLC analysis as described in the main text. Asterisks indicate the genes with significant differences in transcription levels. Fig. S2. Q100TAG mutation in the yifE gene promotes putrescine production. Extracellular concentration of putrescine of AI32 (pQE-80L/MG1655, open diamond) and AI33 (pQE-80L::yifEQ100TAG/MG1655, closed square) cultured at 37°C in minimal M9 supplemented with 0.2% glucose. 100 μg/mL of ampicillin was added to maintain the plasmids. When the OD600 reached 0.5, the 0.5 mM IPTG was added. Fig. S3. The presence of hdfR had a slight, if any, effect on the increase in extracellular putrescine concentration by YifEQ100TAG. (A) Cell growth and (B) extracellular concentration of putrescine of AI32 (pQE-80L/MG1655, open circle), AI33 (pQE-80L::yifEQ100TAG/MG1655, closed triangle), and KT218 (pQE-80L::yifEQ100TAG/ MG1655 but ΔhdfR::FRT-kanR-FRT, closed square) cultured at 37°C in LB supplemented with 100 μg/mL of ampicillin. When the OD600 reached 0.4, 0.02 mM IPTG was added. [file 13568_2021_1330_MOESM1_ESM.docx]

**Supplement**

**Submit to: AMB Express**

**Title: Improvement of putrescine production through the arginine decarboxylase pathway in *Escherichia coli* K-12**

**Running title: Improvement of putrescine production in *Escherichia coli* K-12**

**By-line:** Kullathida Thongbhubate, Kanako Irie, Yumi Sakai, Akane Itoh, and Hideyuki Suzuki**^#^**.

**Affiliation:** Division of Applied Biology, Kyoto Institute of Technology, Matsugasaki, Sakyo-Ku, Kyoto 606-8585, Japan

**#Correspondence**: Hideyuki Suzuki; Fax: +81-75-724-7766; E-mail: hideyuki@kit.ac.jp

This supplement describes the identified mutation in the *yifE* gene as the cause of the increased extracellular putrescine concentration and shows the list of primers used in this study.

**Comparison of the extracellular putrescine concentration among the strains of the Keio collection under anaerobic conditions**

We previously reported that the intake of arginine and bifidobacterium increased the polyamine concentration in the colon (Kibe et al. 2014), although the bifidobacterium cannot synthesize putrescine. As intestinal bacteria are the main source of polyamine in the colon, its concentration in the colon is affected by the rates of biosynthesis, catabolism, and intake and uptake of polyamine by the gut microbiota. We looked for the putrescine exporter in *Escherichia coli* K-12 and identified the SapBCDF transporter as a novel putrescine exporter (Sugiyama et al. 2016). In that study, we grew *E. coli* under aerobic conditions. As the atmosphere of the colon is anaerobic, we looked for a gene that affects the extracellular putrescine concentration under anaerobic conditions in the presence of arginine. Strain MG1655 was grown in M9 glucose medium with and without the addition of 0.2% arginine at 37^o^C under anaerobic conditions. The transcription levels of genes were compared between these two conditions by DNA microarray (data not shown). Twelve genes whose products were annotated as transporters and had significant differences in transcription levels were selected (Fig. S1). The strains of Keio collection deleted these genes were obtained from the National Institute of Genetics, Japan, and their strain numbers are shown in Fig. S1. The additional 15 strains of the Keio collection with deleted genes that encode transporters of amino acids or amines had no differences in transcription levels by DNA microarray. Strain ME9062 is the parental strain of Keio collection strains. As shown in Fig. S1, the extracellular putrescine concentration of strain JW0484 with Δ*ybbA*::FRT-*kan*^R^-FRT was approximately double that of strain ME9062.

**Genome sequence analysis of strains JW0484 and ME9062**

As YbbA was annotated as an ABC transporter whose substrate was unknown (Moussatova et al. 2008), we expected YbbA to encode a subunit of a new putrescine importer. P1*vir* phage was grown on strain JW0484 and MG1655 was transduced with the phage lysate to obtain a Kan^R^ transductant that should be Δ*ybbA*::FRT-*kan*^R^-FRT. However, the extracellular putrescine concentration of the transductant and the parental MG1655 was the same. We predicted that the second mutation in strain JW0484 increased the extracellular putrescine concentration. Genomic DNA of strains JW0484 and ME9062 was isolated, and their DNA sequences were compared. The DNA sequence of strain MG1655 in the database was used as a reference sequence. In addition to the mutations in strain ME9062, additional mutations were found in *intA*, *fbaA*, *dgoR*, *yifE*, and *lexA* in the genome of strain JW0484. The Kan^R^ cassette was excised from strain JW0484 by pCP20 as described previously (Datsenko and Wanner 2000), and the obtained Kan^S^ strain (YS25) was used as a host strain of the following transduction. For example, *yifE* and *yifB* genes are located at 85.06 and 85.07 min, respectively, on the *E. coli* K-12 genome. P1*vir* phage was grown in strain JW3738 (Δ*yifB*::FRT-*kan*^R^-FRT strain of the Keio collection) and strain YS25 was transduced with this lysate to obtain Kan^R^ transductants. We expected 98.5% of the transductants to be *yifE*^+^. The extracellular putrescine concentrations of four obtained transductants were as low as that of strain ME9062. The other four newly found mutations in strain JW0484 were reverted similarly, but the extracellular putrescine concentrations of the obtained transductants were almost the same as that of strain YS25. Therefore, we concluded that mutation of the *yifE* gene caused the high extracellular putrescine concentration. According to the DNA sequence of the *yifE* mutant, Gln100 was mutated to a TAG stop codon, resulting in a 13-residue shorter protein than the wild-type YifE.

**Comparison of transcription level of *hdfR* and *gltB* between strains ME9062 and JW0484**

Although strains ME9062 and JW0484 were grown under aerobic conditions, the extracellular concentration of JW0484 was notably higher than that of ME9062. Therefore, JW0484 and ME9062 were grown in M9 medium containing 0.2% glucose supplemented with 0.2% arginine, and grown until the OD_600_ reached 0.3 to 0.4. In total, 1x 10^9^ cells were collected and mixed with 400 μL of lysozyme in TE buffer (pH 8.0, 1 mg/mL). Then, total RNA was isolated from the cells using the RNeasy mini kit (Qiagen; Hilden, Germany) according to the manufacturer's instructions. Samples were digested with DNase I (Qiagen) to remove contaminating genomic DNA. The RNA concentration and quality were evaluated by measuring the AB_260_ and the 260 nm/280 nm ratio, respectively.

To generate cDNA, total RNA (1 μg) was reverse-transcribed using the iScript cDNA Synthesis Kit (Bio-Rad; Hercules, CA), and 2 units of Tth RNaseH (Toyobo; Osaka, Japan) was added to a final volume of 20 μL and incubated at 37°C for 20 min. cDNA was stored at -20°C until use. Real-time (RT-PCR) was performed using the Power SYBR Green PCR Master Mix (Applied Biosystems; Waltham, MA) and StepOne Real-time PCR Systems (Applied Biosystems). The sequences of primers are listed in Table S3. After amplification, melting curves were obtained to ensure that all PCR products were amplified. StepOne Software v2.1 was used to quantify the relative mRNA levels of the target genes after normalization against the housekeeping gene *dnaE*.

The DNA-binding transcriptional dual regulator *hdfR* is assumed to be a target of *yifE* because it is located adjacent to *yifE* on the *E. coli* genome. We performed RT-PCR to examine whether the expression level of *hdfR* is affected in JW0484. The expression level of *hdfR* was higher in JW0484 than in its parental strain ME9062 (data will be published elsewhere). This suggests that wild-type *yifE* functions as a transcriptional repressor of the *hdfR* gene. Moreover, the expression level of the *gltB* gene, which is known as a regulatory target of *hdfR*, increased in JW0484. Thus, in JW0484, the mutated *yifE* gene does not repress *hdfR*, and *gltB* gene expression increases. This suggested that glutamate synthesis is promoted in JW0484, possibly resulting in the increase in putrescine synthesis and its excretion from the cell. However, additional studies are needed to confirm this hypothesis.

While addressing this issue, the *yifE* gene was reported to encode macrodomain Ori protein by Valens et al. (2016). How macrodomain Ori protein promotes putrescine excretion remains unknown.

**The *yifE*^Q100TAG^ mutation increases the extracellular putrescine concentration**

According to our previous RT-PCR results, it is still unclear whether the mutated *yifE* gene in JW0484 increases putrescine production. To clarify the effects of *yifE* mutation on putrescine production, the *yifE*^Q100TAG^ was introduced into a vector (pQE-80L). Then, the parental strain MG1655 was transformed with the plasmid with *yifE*^Q100TAG^, resulting in strain AI33. The strain AI32, which is MG1655 containing empty vector (pQE-80L), was used as a control. The extracellular concentration of putrescine after 30 h increased 5 folds in AI33 compared with AI32 (Fig. S2). Thus, the point mutation in the *yifE* gene had a positive effect on putrescine production even in the presence of the wild-type *yifE* gene on the genome.

**Putrescine production is affected by the YifE^Q100TAG^ mutant, but little by the increase in HdfR**

The *yifE*^Q100TAG^ mutant increased the extracellular concentration of putrescine and the transcription level of *hdfR* gene. However, it was still unclear whether the increase of transcription level of *hdfR* by *yifE*^Q100TAG^ mutant directly relates to the increase of the extracellular concentration of putrescine. To answer this question, the strain KT217 was constructed by transducing the Δ*hdfR*::FRT-*kan*^R^-FRT from the Keio gene knockout collection (Baba et al. 2006) to strain MG1655. pAI07, which is pQE-80L contains *yifE*^Q100TAG^, was introduced to KT217 and strain KT218 was obtained. To evaluate the effect of the presence and the absence of HdfR on putrescine-production enhancing effect of YifE^Q100TAG^, the strains AI32 (pQE-80L/ MG1655), AI33 (pQE-80L::*yifE*^Q100TAG^/ MG1655), and KT218 (pQE-80L::*yifE*^Q100TAG^/ MG1655 but Δ*hdfR*::FRT-*kan*^R^-FRT) were grown in LB medium supplemented with 100 μg/mL of ampicillin. As shown in Fig. S3(A), the cell growth profiles of three strains were almost the same. AI32 exhibited the lowest extracellular putrescine concentration than AI33 and KT218. And KT218 exhibited rather lower extracellular putrescine concentration than AI33 (Fig. S3(B)). These results suggest that the increase of extracellular putrescine production by *yifE*^Q100TAG^ can occur without *hdfR* gene.

**References**

Baba T, Ara T, Hasegawa M, Takai Y, Okumura Y, Baba M, Datsenko KA, Tomita M, Wanner BL, Mori H (2006) Construction of *Escherichia coli* K-12 in-frame, single-gene knockout mutants: the Keio collection. Mol Syst Biol 2:2006-0008. https://doi.org/10.1038/msb4100050

Datsenko KA, Wanner BL (2000) One-step inactivation of chromosomal genes in *Escherichia coli* K-12 using PCR products. Proc Natl Acad Sci U S A 97:6640-6645. https://doi.org/10.1073/pnas.120163297

Kibe R, Kurihara S, Sakai Y, Suzuki H, Ooga T, Sawaki E, Muramatsu K, Nakamura A, Yamashita A, Kitada Y, Kakeyama M, Benno Y, Matsumoto M (2014) Upregulation of colonic luminal polyamines produced by intestinal microbiota delays senescence in mice. Sci Rep 4:1-11. https://doi.org/10.1038/srep04548

Moussatova A, Kandt C, O'Mara ML Tieleman DP (2008) ATP-binding cassette transporters in *Escherichia coli*. Biochim Biophys Acta 1778:1757-1771. https://doi.org/10.1016/j.bbamem.2008.06.009

Sugiyama Y, Nakamura A, Matsumoto M, Kanbe A, Sakanaka M, Higashi K, Igarashi K, Katayama T, Suzuki H, Kurihara S (2016) A novel putrescine exporter SapBCDF of *Escherichia coli*. J Biol Chem 291:26343-26351. https://doi.org/10.1074/jbc.M116.762450

Valens M, Thiel A, Boccard F (2016) The *MaoP*/*maoS* Site-Specific System Organizes the Ori Region of the *E. coli* Chromosome into a Macrodomain. PLoS Genet 12:e1006309. https://doi.org/10.1371/journal.pgen.1006309

**Table S1 Primers used for strain construction**

| Primer Sequence |
| --- |
| delta-ygjG F gcgcaatccctgcaatacttaaatcggtatcatgtgatacgcgagcctcc  ggagcatattgtgtaggctggagctgcttc  delta-ygjG R gggcgtacgcgtcgtataaaaagatcggatggcgacgtcgtatcgccatc  cgatttgataattccggggatccgtcgacc  patAprimer_Fupstream400bp cccgttatatggttgttaacttc  patAprimer_Rdownstream402bp cccgtaaaattttaccctaagggag  potF-up acaacttttgttcgtttgttaacgaactttcagaaggaaagagatattcc  ggggatccgtcgacc  potI-down gacatgcgcggcattatgtagccaggttggcaaattttagtgtcttgtag  gctggagctgcttcg  pKD13-priming site1 gtgtaggctggagctgcttc  delta ygjG kakunin R atggggaaaacggtggtggt  potF up comf ttcttacaccgcgccgataa  potI down comf aaaatcccaacgtttcgtct |

**Table S1 Primers used for strain construction (cont)**

| Primer Sequence |
| --- |
| F_clo_IR_hdfR cccatgacgagagaatccacgtggataatg  Colony PCR hdfR 279bp gcagtaagccggtcataaaac |

**Table S2 Primers used for plasmid construction**

| Primer Sequence |
| --- |
| EcoRI_SD_ATG_argA_F cccgaattcattaaagaggagaaattaactatggtaaaggaacgtaaaaccgag  HindIII_argA_R cccaagcttaccctaaatccgccatcaacac  argA_Y19C_F ggattccgccattcggttccctgcatcaatacccaccggggaaaaacg  argA_Y19C_R cgtttttccccggtgggtattgatgcagggaaccgaatggcggaatcc  KpnI_SD_GTG_argA_F cccggtaccgaggagaaattaactgtggtaaag  KpnI_SD_ATG_argA_Y19C F cccggtaccgaggagaaattaactatggtaaag  HindIII_argA_cloning R new cccaagcttttaccctaaatccgccatca  pQE-80L speA cloning F(EcoRI) cccgaattcattaaagaggagaaattaactattctgacgacatg  speA cloning R(SphI) cccgcatgcttactcatctt  long SphI SD speB cloning F cccgcatgcgaggagaaattaactatgagcaccttaggtcatca  long speB cloning R(KpnI) cccggtaccttactcgccctttttcgccg  argA_ATG_Y19C_cloningR(HindIII) cccaagcttttaccctaaat  SacI_argA_ATG_F cccgagctcgaggagaaattaactatggtaaag  SalI_argA_Rev cccgtcgacttaccctaaatccgccatcaacac |

**Table S2 Primers used for plasmid construction (cont.)**

| Primer Sequence |
| --- |
| HindIII_Xbal_AT_StuI_AT_SalI_rrnB T1 N cccaagctttctgaataggcctatgtcgacgcaaatattatacgcaaggc  NdeI_rrnB T1C ccccatatgggcggatttgtcctactcag  lacIq N_SphI cccgcatgctggtgcaaaacctttcgcgg  speA cloning R(SphI) cccgcatgcttactcatctt  NdeI_dXbalI_lacIq_N ccccatatgtcatgcgcacccgtggccag  EcoRI_ope_lacI^q^promoter_SphI_comp cccgaattcaattgttatccgctcacaattcgggcgctatcatgccataccgcgaaagg  ttttgcaccagcatgc  NdeI_5978 tgacatatgcggtgtgaaataccgc  SphI_lacIq1p primer gcggcatgctgttgacaccacctttcgcggtatggcatgatagcgc  SphI_lacIp_pSH1807_new gcggcatgctggcgcaaaacctttcgcggtatgg  carA N’ HindIII cccaagcttgaggagaaattaactatgattaagtcagcgctattg  carB C’ SalI-StuI-Xbal cccgtcgacataggcctattctagattatttgatctgtgcgtgcatttc |

**Table S2 Primers used for plasmid construction (cont.)**

| Primer Sequence |
| --- |
| glnA N’ Xbal ccctctagagaggagaaattaactatgtccgctgaacacgtactg  glnA C’ StuI cccaggcctttagacgctgtagtacagctc  pQE-80LspeCcloningf (EcoR1) cccgaattcattaaagaggagaaattaactatgaaatcaatgaat  speCcloningr(Sac1) cccgagctcttacttcaacacataaccgta  Nhe1 T5P lacO cloning cccgctagccctttcgtcttcacctcgag  Lambda termi Nco1 R cccccatggccaagctagcttggattctc |

**Table S3 Primers used for Realtime PCR**

| Primer Sequence |
| --- |
| dnaE-RT-Fw atgtcggaggcgtaaggct  dnaE-RT-Rv tccagggcgtcagtaaacaa  hdfR-RT-Fw ccgccacagaaacaatatcc  hdfR-RT-Rv ggtatgcgccacctccttac  gltB-RT-Fw tgatgtccccactaacgaagg  gltB-RT-Rv ggcgggcgataaacaga |

**Fig. S1 Comparison of extracellular putrescine concentration per OD_600_ among strains of the Keio collection.** M9 glucose medium (60 mL in 100-mL Erlenmeyer flask with stirrer bar) was inoculated with the pre-culture at the initial OD_600_ of 0.06. The flasks were set in the 7-L rectangular anaerobic jar with 2 AnaeroPack-Anaero sachets (Mitsubishi Gas Chemical; Tokyo, Japan) to form an anaerobic environment and the jar was set on the stirrer in the microbiological incubator. The culture medium was stirred at 150 rpm and kept at 37^o^C. After 10 h of incubation, the OD_600_ of the culture was measured and 0.4 mL of the culture was centrifuged. Thirty μL of 100% (w/v) TCA was mixed with 0.3 mL of the supernatant, filtrated through the membrane filter, and then subjected to HPLC analysis as described in the main text. Asterisks indicate the genes with significant differences in transcription levels.

**Fig. S2 Q100TAG mutation in the *yifE* gene promotes putrescine production.**

Extracellular concentration of putrescine of AI32 (pQE-80L/MG1655, open diamond) and AI33 (pQE-80L::*yifE*^Q100TAG^/MG1655, closed square) cultured at 37°C in minimal M9 supplemented with 0.2% glucose. 100 μg/mL of ampicillin was added to maintain the plasmids. When the OD_600_ reached 0.5, the 0.5 mM IPTG was added.

**(A)**

**(B)**

**Absorbance (OD_600_)**

**Cultivation time (h)**

**Cultivation time (h)**

**Putrescine concentration (µM)**


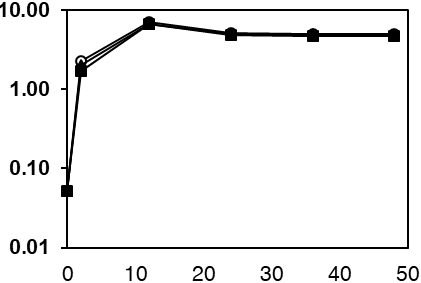

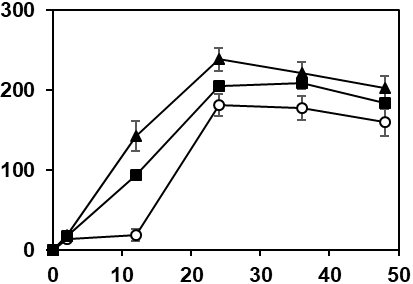


**Fig. S3 The presence of *hdfR* had a slight, if any, effect on the increase in extracellular putrescine concentration by YifE^Q100TAG^**

(A) Cell growth and (B) extracellular concentration of putrescine of AI32 (pQE-80L/MG1655, open circle), AI33 (pQE-80L::*yifE*^Q100TAG^/MG1655, closed triangle), and KT218 (pQE-80L::*yifE*^Q100TAG^/ MG1655 but Δ*hdfR*::FRT-*kan*^R^-FRT, closed square) cultured at 37°C in LB supplemented with 100 μg/mL of ampicillin. When the OD_600_ reached 0.4, 0.02 mM IPTG was added.
